# The current utilization of the patient-reported outcome measurement information system (PROMIS) in isolated or combined total knee arthroplasty populations

**DOI:** 10.1186/s43019-023-00177-3

**Published:** 2023-01-19

**Authors:** Puneet Gupta, Natalia Czerwonka, Sohil S. Desai, Alirio J. deMeireles, David P. Trofa, Alexander L. Neuwirth

**Affiliations:** Department of Orthopaedic Surgery, Columbia University Irving Medical Center, 622 West 168th Street, New York, NY 10032 USA

**Keywords:** Knee, Knee arthroplasty, Total knee replacement, Patient-reported outcome measures

## Abstract

Patient reported outcome measures (PROMs) are essential for clinical research and patient-centric care because they allow us to capture patient perspectives on their health condition. In knee arthroplasty, PROMs are frequently used to assess the risks and benefits of new interventions, surgical approaches, and other management strategies. A few examples of PROMs used in total knee arthroplasty (TKA) include the Oxford Knee Score (OKS), Knee Injury and Osteoarthritis Outcome Score (KOOS), and the Forgotten Joint Score (FJS) (collectively referred to as “legacy” PROMs). More recently, attention has been brought to another PROM called the Patient-Reported Outcomes Measurement Information System (PROMIS). PROMIS was developed by the National Institute of Health (NIH) and has over 300 domains assessing various aspects of patient health, including pain, physical function, and mental health. With the use of PROMIS increasing in TKA literature, there is a need to review the advancements being made in understanding and applying PROMIS for this population. Thus, the purpose of this study is to provide insight on the utilization, advantages, and disadvantages of PROMIS within the field of knee arthroplasty and to provide a comparison to legacy PROMs.

## Introduction

Patient-reported outcome measures (PROMs) were developed to gain a better understanding of the patient’s perspective to facilitate patient-centric care and standardize outcomes research. PROMs allow us to capture many different aspects of a patient’s health from the patient directly, including their pain, physical function, functional status, general perceptions or attitudes, emotional health, and more. PROMs are used in all areas of medicine and surgery, and many have been developed that are specific to a certain population, disease, or procedure [1–4]. In orthopedics, many PROMs have been developed and validated within all subspecialties [5]. For example, the Foot and Ankle Disability Index is used in foot and ankle surgery, the Boston Carpal Tunnel Questionnaire is used in hand and upper extremity surgery, and the Neck Disability Index is used in spine surgery [5]. In hip and knee arthroplasty, a few commonly used PROMs include the Oxford Hip Score (OHS), the Knee Society Clinical Rating System (KSCRS), and the Western Ontario and McMaster Universities Osteoarthritis Index (WOMAC) score [6]. Despite the development of these PROMs, there remain limitations with these “legacy” PROMs.

**Fig. 1:**
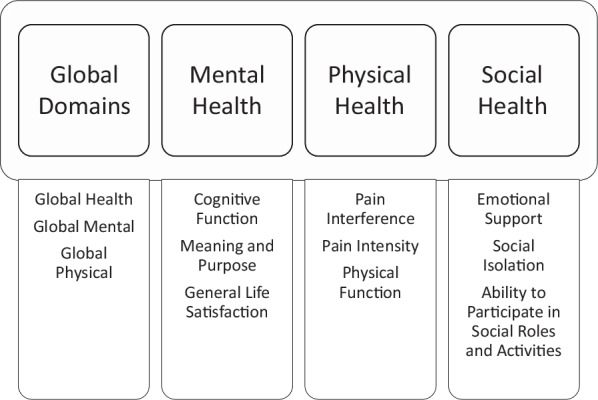
PROMIS domains and examples of measures available

In 2004, the National Institute of Health (NIH) established the Patient-Reported Outcomes Measurement Information System (PROMIS) to standardize the collection of PROs. PROMIS is “a set of person-centered measures that evaluates and monitors physical, mental, and social health in adults and children [7].” PROMIS has over 300 measures of health, with domains including pain interference (PI), physical function (PF), depression (DEP), fatigue, anxiety, sleep disturbance, and more (Fig. 1) [8]. Many institutions and researchers have now integrated PROMIS into their clinical research and workflow, resulting in over 1000 publications and more than 100 NIH grants [8, 9]. The purpose of this manuscript is to provide insight on the utilization, advantages, and disadvantages of PROMIS within the field of knee arthroplasty, and to provide a comparison to legacy PROMs.

## Overall advantages of PROMIS versus legacy instruments

Given the increasing utilization of PROMIS for evaluating and reporting PROs, it is important to explore its associated advantages relative to legacy instruments. The advantages of PROMIS are applicable not just to knee arthroplasty, but more broadly, to all areas of medicine and clinical research.

One major advantage of PROMIS is that it can be deployed on multiple platforms (written form, mobile, and web) in two distinct formats [short form (SF) and computerized adaptive testing (CAT)]. SFs typically consist of a static set of 4–10 questions. CATs were founded on item response theory and allow for dynamic testing by selecting the next question from a large item bank based off a patient’s prior response, usually leading to a total of 4–8 questions [8]. Such versatility in PROMIS architecture improves feasibility of adoption by institutions of all types, facilitates integration into an organization’s clinical research flow, and supports robust and consistent data collection.

A second advantage of PROMIS is that it is highly time efficient compared with other PROMs [10–13]. In a retrospective study of 2170 patients undergoing hip arthroscopy, Browning et al. aimed to evaluate the time to completion for PROMIS questionnaires compared with legacy PROs [10]. The median time to completion for PROMIS questionnaires was 37 s for PI, 43 s for DEP, and 46 s for PF. In contrary, the median time to completion (min:s) was much longer for modified Harris Hip Score (mHHS) (1:29), Hip Outcome Score (HOS) (3:58), and International Hip Outcome Tool (iHOT-12) (2:11). These findings of a quicker completion time for PROMIS in hip arthroscopy patients compared with legacy PROs is consistent with other orthopedic studies as well [10–13].

A third advantage of PROMIS is that it is standardized and reports all outcomes with standardized *T*-scores. Having a common set of measures and metrics can help accelerate research advances by improving the translatability of patient-centered data. Systematic reviews and meta-analyses could be significantly strengthened by use of this standardized metric. Although CATs use item response theory and thus can result in different sets of questions between individuals, the precision in obtaining the final score is high and equal between individual sets [14–16].

A fourth advantage of PROMIS is that translations are available in over 40 different languages, allowing for broad adoption and use in many different populations. A fifth advantage of PROMIS is that it can be used in patients of all ages and includes adult domains, self-reported pediatric domains, and parent proxy or parent-reported domains.

## Overall disadvantages of PROMIS versus legacy instruments

Despite the aforementioned advantages of PROMIS compared with legacy instruments, there are a few disadvantages to be aware of. One is the costs associated with integrating and implementing PROMIS in an orthopedic system, although this financial burden may vary based on practice setting. A recent study found that the costs associated with establishing a PROMIS-based registry at a single surgeon’s sports medicine clinic was estimated to be US$2045 and had a monthly maintenance cost of US$1000 [17]. These costs may be mitigated by utilization of pen and paper SF PROMIS questionnaires. In addition to the costs, certain electronic health records (EHRs) may not allow or may have more barriers for integrating PROMIS. Furthermore, new features and updates to domains in PROMIS are continually being made, and so this dynamic platform may lead to difficulties with longitudinal data collection or comparing and contrasting to previously collected data.

A final theoretical limitation is that disability or injury in one part of the body may obscure the outcome data of interest from a different part of the body, due to the broad nature of certain PROMIS domains and their nonspecificity for a particular anatomical region. For example, a patient with an Achilles tendon tear may have decreased PF, which would obscure postoperative outcome data following TKA. This limitation may be mitigated by exclusion of patients from PROMIS research studies that have potentially confounding comorbidities, or by using PROMIS domains that are more specific (e.g., PROMIS PF Upper Extremity for upper extremity ailments).

## PROMIS validation and comparisons with legacy instruments: knee arthroplasty

In several areas of knee arthroplasty, PROMIS CAT and SF forms have been evaluated for validation and comparison with legacy instruments (Table 1). Several domains of PROMIS have been compared, including PF, PI, and pain intensity. Khalil et al. aimed to investigate the relationship between Knee Injury and Osteoarthritis Outcome Score for joint replacement (KOOS JR), PROMIS Global Physical Health (GPH) and PROMIS Global Mental Health (GMH) in knee arthroplasty patients, excluding revision surgery patients [18]. The PROMIS Global Health (GH) form has ten items, of which four are scored into a physical health summary score (GPH), four are scored into a mental health summary score (GMH), and two that are not used for either GPH or GMH. In this retrospective study of 875 patients, postoperative PROMIS GPH and KOOS JR scores were significantly correlated with each other. PROMIS GMH was not responsive, but the PROMIS GPH score was moderately responsive at 1 and 3 months and excellently responsive at 6 and 12 months. KOOS JR demonstrated excellent responsiveness at all time points. Overall, PROMIS forms were found to be correlated with KOOS JR scores, but KOOS JR scores were more responsive in the early postoperative period [18].

**Table 1:** PROMIS validation studies in knee arthroplasty

Author and date	Cohort	PROMIS instruments	Legacy instruments
Padilla et al. 2019	Knee pain and subset of knee arthroplasty patients	PROMIS PF PROMIS PI PROMIS pain intensity	KOOS JR
Heng et al. 2021	Primary TKA	PROMIS PF	KOOS functional in activities of daily living
Tang et al. 2022	Primary TKA	PROMIS PF	KOOS PF
Austin et al. 2019	TKA	PROMIS global physical health PROMIS global mental health	Modified single assessment numerical evaluation (M-SANE) score
Shim et al. 2019	Primary TKA for osteoarthritis	PROMIS global physical health PROMIS global mental health	EuroQol five-dimension (EQ-5D) OKS
Givens et al. 2018	Candidates for primary TKA	PROMIS PF	Timed Up and Go (TUG) test
Lawrie et al. 2021	Bundled payment for care improvement (BCPI) primary TKA patients	PROMIS PF PROMIS PI PROMIS Depression	–
Kortlever et al. 2020	Knee pain	PROMIS PF	KOOS JR
Hung et al. 2018	Primary or revision THA or TKA	PROMIS PF	HOOS JR KOOS JR

*PROMIS* patient-reported outcomes measurement information system, *PF* physical function, *PI* pain interference, *TKA* total knee arthroplasty, *KOOS JR* Knee Injury and Osteoarthritis Outcome Score for joint replacement, *OKS* Oxford Knee Score

Similarly, Padilla et al. aimed to compare PROMIS CAT PF, PI, and pain intensity to previously validated KOOS JR forms in knee pain patients and a subset of knee arthroplasty patients [19]. The authors analyzed 1620 patients and 2133 questionnaires from a single institution with knee-related pain (124 knee arthroplasty patients). KOOS JR was found to have moderate-to-strong correlations (*r*-values ranging from 0.56 to 0.71) with each of the PROMIS CAT PF, PI, and pain intensity questionnaires. When looking at specifically knee arthroplasty patients, the strength of the correlations remained moderate to strong. This large study supports the validity of these PROMIS domains in knee pain and knee arthroplasty patients [19].

Furthermore, Heng et al. aimed to see if KOOS function in Activities of Daily Living (ADL) could be linked to PROMIS PF [20]. In this retrospective study, 1003 primary TKA patients with a mean age of 67 years were identified, of which 98% had a diagnosis of primary osteoarthritis. The authors successfully created a crosswalk of equivalent scores between both KOOS ADL and PROMIS PF to allow for conversion of scores in both directions. This creation of a crosswalk table can help store and compare PROM data and allow clinicians and researchers greater flexibility in the scores they decide to use for a primary TKA patient population with predominantly osteoarthritis [20].

Similarly, Tang et al. aimed to investigate whether KOOS PF SF could be linked to PROMIS PF SF scores using five linking approaches [21]. In this retrospective study, data from 3667 patients who were under consideration for primary TKA was used. This population included a mix of nonsurgical, preoperative, and postoperative patients from academic and community hospitals. The observed *T*-scores and linked *T*-scores had high correlations (*r* ≥ 0.78) and minimal mean differences for all linking methods, with the Stocking–Lord approach chosen as best. A crosswalk table to convert KOOS PF SF raw and summary scores to PROMIS PF *T*-scores was successfully developed and validated by the authors. This study supports integration of the PROMIS PF domain into a clinical and research workflow by providing reliable conversions between PROMIS PF and KOOS PF SF scores [21].

Austin et al. compared PROMIS GPH and GMH with a one-question, modified single assessment numerical evaluation (M-SANE) score in patients undergoing TKA [22]. In this retrospective study of 217 patients from a single institution, PROMIS GPH and M-SANE had Spearman’s Rho coefficients of 0.28, 0.40, and 0.65 (all *p* < 0.001) at preoperative, immediate follow-up (1–90 days postoperatively), and extended follow-up (270–365 days postoperatively), respectively. PROMIS GMH and M-SANE were weakly correlated preoperatively and at all follow-ups [Spearman Rho coefficients ≤ 0.31 (all *p* < 0.001)]. PROMIS GPH had extremely low floor and ceiling effects at all time points (max floor effect < 0.1% and max ceiling effect of 1.6%). PROMIS GMH also had low floor effects (< 0.1% max) and low ceiling effects (7.2% max) at all time points, which were lower than M-SANE. Moreover, PROMIS GPH was found to be more responsive [standardized response mean (SRM) of 0.61] from the immediate follow-up to extended follow-up versus M-SANE (SRM 0.34), although M-SANE was more responsive from preoperative to immediate follow-up and from preoperative to extended follow-up [22].

Shim et al. aimed to compare the responsiveness of PROMIS SF GPH, GMH, and EuroQol five-dimension (EQ-5D) in patients undergoing primary TKA for osteoarthritis [23]. Both PROMIS GPH and EQ-5D demonstrated high responsiveness (SRM > 0.7) at all time points, with PROMIS GPH having relatively higher SRM values (range 1.06–1.20) than EQ-5D (range 0.72–0.87). PROMIS GMH did not demonstrate statistically significant responsiveness at any time point. External responsiveness was evaluated by correlating changes in scores with changes in the joint-specific Oxford Knee Score (OKS) [23]. PROMIS GPH was also found to have relatively better external responsiveness, as the change in PROMIS GPH scores had a greater correlation coefficient (0.57) with change in OKS score at 6 months relative to EQ-5D (0.51). Additionally, PROMIS GPH showed good discrimination ability [area under the receiver operating characteristic curve (AUC) of 0.82] between patients achieving OKS score Minimum Clinically Important Difference (MCID) (> 5 points) and those that did not. Overall, this study showed that PROMIS GPH, but not GMH, has greater internal and external responsiveness relative to EQ-5D and is sensitive to clinically significant change in patients undergoing primary TKA for OA [23].

Givens et al. aimed to assess whether PROMIS PF CAT can be used as a surrogate for the Timed Up and Go (TUG) test for TKA candidates [24]. The authors found that PROMIS PF CAT was not a good surrogate for TUG, as there was only a moderate correlation (*r* = −0.43) between both. However, TUG was the best predictor of PROMIS PF CAT relative to BMI, smoking status, and the numeric pain rating scale (NPRS). Despite this, this study provides evidence that the TUG test and PROMIS PF are not well correlated and should not be used interchangeably in a population of TKA candidates [24].

PROMIS is being evaluated and validated in specific knee arthroplasty populations. Lawrie et al. aimed to assess PROMIS feasibility, responsiveness, and scores in Bundled Payment for Care Improvement (BCPI) primary TKA patients [25]. In this retrospective study of 172 patients, both BCPI and non-BCPI patients had significant improvements in PROMIS PF, PI, and DEP domains between the preoperative and 1-year follow-up time points with no significant difference in MCID achievement rates. No ceiling effects were seen in either group. Floor effects were seen in both groups for PROMIS DEP (38% for each) and PI (20% in BCPI, 14% in non-BCPI) at 1-year follow-up. Patient PROMIS assessment refusal rate was 2% and the average time to answer all three CATs was 140 s [25]. This study showed that PROMIS PF, PI, and DEP scores are responsive in the TKA population irrespective of BCPI enrollment, although PI and DEP use may be more limited due to their larger floor effects [25].

Kortlever et al. evaluated whether there was any correlation with PROMIS PF and KOOS JR in patients with knee pain, although specific diagnoses were not specified [26]. The authors conducted a cross-sectional study of 94 patients from two arthroplasty practices and one practice including arthroplasty and trauma surgeons. Both questionnaires were completed in under 60 s. PROMIS PF and KOOS JR were found to have a high correlation coefficient of 0.74. No ceiling or floor effects were found for PROMIS PF, and KOOS JR had only a mild floor effect of 3.4% and a ceiling effect of 1.1%. This highlights the similarity of PROMIS PF and KOOS JR and indicates that the two PROMs may be interchangeable in patients with knee pain [26].

## PROMIS utilization in knee arthroplasty studies

In the setting of increasing evidence of the validity and correlation with legacy instruments displayed by PROMIS, knee arthroplasty researchers are now utilizing PROMIS to evaluate patient outcomes. Ingall et al. aimed to evaluate the risks of preoperative opioid use on PROMs following revision TKA [27]. In a retrospective, propensity score-matched analysis of 330 patients, opioid users were found to have significantly lower postoperative PROMIS GPH (*p* < 0.001) and PROMIS GMH scores (*p* < 0.001) [27]. Similarly, Klemt et al. compared PROMs following single-stage and two-stage revision TKA for chronic periprosthetic joint infections [28]. Patients undergoing single-stage revision TKA had significantly higher PROMIS GPH (*p* = 0.01), GMH (*p* = 0.02), and PF (*p* < 0.01) scores postoperatively compared with two-stage revision patients [28].

Kagan et al. conducted a prospective observational study of 91 patients to understand the typical recovery in PROMIS PF CAT and PROMIS PI CAT after primary TKA [29]. For PF, all postoperative *T*-scores were significantly greater than preoperative *T*-scores (*p* < 0.001), except for the 6-week postoperative mark (*p* = 0.410). For PI, all postoperative *T*-scores were significantly reduced compared with preoperative scores (*p* ≤ 0.001). Clinically important differences between preoperative and postoperative visits for both PF and PI were first seen at the 3-month postoperative visit (*p* < 0.001), and a majority of the PF improvement (63%) and PI reduction (68%) were seen by 3 months [29]. Such findings allow for a better discussion between surgeons and patients regarding expected postoperative recovery following TKA [29].

Christensen et al. aimed to understand how preoperative physical and psychological health influences PROMIS scores 1 year following primary unilateral TKA in young patients (≤ 65 years) [30]. In an analysis of 65 patients, poor preoperative physical activity (low University of California at Los Angeles activity rating scale score) (*p* < 0.01), greater Charlson comorbidity index (CCI) (*p* = 0.01), and worse PI *T*-scores (*p* = 0.03) were found to be significant risk factors for worse 1-year postoperative PROMIS PF *T*-scores. Additionally, a greater CCI was found to result in worse patient satisfaction scores 1 year postoperatively (*p* < 0.01) [30].

Darrith et al. aimed to identify patient factors that influence postoperative PROMIS GH (GPH and GMH) scores and the prognostic utility of preoperative PROMIS GH (GPH and GMH) scores in predicting postoperative improvement following primary TKA [31]. A retrospective cohort study of 872 patients undergoing unilateral primary TKA was performed. No patient demographics, comorbidities, or laboratory values were associated independently with pre- or postoperative PROMIS GPH scores following multivariate analysis (*p* > 0.05). Only preoperative PROMIS GPH was independently associated with postoperative PROMIS GPH scores at 1 month (*p* < 0.001), 6 months (*p* < 0.001), and 12 months (*p* = 0.020). Additionally, only preoperative PROMIS GPH predicted postoperative MCID achievement at all time points (*p* < 0.01 at 1 month, *p* < 0.01 at 3 months, *p* = 0.022 at 6 months, *p* = 0.006 at 1 year), highlighting its potential prognostic utility in primary TKA patients [31].

## PROMIS validation, comparison to legacy instruments, and utilization in combined hip and knee arthroplasty populations

Hung et al. conducted a study to compare the responsiveness of PROMIS PF CAT to HOOS JR and KOOS JR at a joint reconstruction practice [32]. In this study, there were 983 patients of which 87% underwent primary or revision THA or TKA. All three PROMs were highly responsive at the 3-month, more than 3-months, and more than 6-months follow-up time points (*p* < 0.05). Although all PROMs had high effect sizes, PF had the highest effective size at all follow-up periods, with a peak effect size of 1.20 at the 6-month follow-up. All three PROMs also had high SRMs (> 1.0) for all follow-up periods. At the 3-month follow-up, PROMIS PF had the highest SRM (1.53) relative to HOOS JR and KOOS JR. This shows that PROMIS PF CAT is highly responsive and comparable to the legacy instruments HOOS JR and KOOS JR in a predominantly joint arthroplasty practice [32].

Horn et al. aimed to assess whether and which PROMIS domains can be used to differentiate patients undergoing THA or TKA from nonsurgical patients [33]. In this retrospective study, a total of 269 total joint arthroplasty (TJA) cases and 545 nonsurgical cases were used and PROMIS scores for eight domains including, PF, PI, DEP, pain intensity, anxiety, sleep disturbance, fatigue, and ability to participate in social roles and activities were collected. After controlling for comorbidities and confounders, only a lower PROMIS PF score was found to be associated with patients undergoing hip or knee TJA (*p* < 0.01). This shows that PROMIS PF could be a useful score to obtain from patients with hip or knee osteoarthritis, as it may help identify and differentiate between patients who are likely to undergo TJA and nonsurgical patients [33].

## Future studies with PROMIS

Further studies are warranted, however, to continue expanding the utility of PROMIS. For example, no study evaluating PROMIS responsiveness and validity in only revision TKA patients has been done. As such, PROMIS needs to continue to be investigated in much larger sample sizes in different subpopulations, including those with traumatic indications (e.g., proximal tibia fracture) and inflammatory indications (e.g., rheumatoid arthritis). Likewise, PROMIS validity and responsiveness should be examined in different racial populations, insurance groups, and geographic regions. Furthermore, PROMIS comparisons to other legacy PROMs are necessary, including the KSCRS and Forgotten Joint Score (FJS).

## Conclusions

Overall, early studies on PROMIS use in knee arthroplasty are promising. They have explored both CAT and SF instruments and several PROMIS domains in varying populations. In several knee arthroplasty and knee pain studies, PROMIS GPH and PF were frequently evaluated and found to be responsive, valid, and correlate well with legacy knee scores. This has even led to the development of crosswalk tables between KOOS ADL and KOOS PF with PROMIS PF. In contrast, PROMIS GMH was often not responsive and did not correlate as well with legacy instruments. Surgeons should be aware of the advancements being made in using PROMIS domains for knee arthroplasty patients. Knee surgeons should consider utilizing PROMIS domains, especially GPH and PF, in their clinical practice.

## Data Availability

Not applicable.
